# Angle-of-Arrival Estimation Using Difference Beams in Localized Hybrid Arrays

**DOI:** 10.3390/s21051901

**Published:** 2021-03-09

**Authors:** Hang Li, Zhiqun Cheng

**Affiliations:** School of Electronics and Information, Hangzhou Dianzi University, Hangzhou 310018, China; zhiqun@hdu.edu.cn

**Keywords:** hybrid array, localized subarrays, angle-of-arrival estimation, mmWave communications

## Abstract

Angle-of-arrival (AoA) estimation in localized hybrid arrays suffers from phase ambiguity owing to its localized structure and vulnerability to noise. In this letter, we propose a novel phase shift design, allowing each subarray to exploit difference beam steering in two potential AoA directions. This enables the calibration of cross-correlations and an enhanced phase offset estimation between adjacent subarrays. We propose two unambiguous AoA estimation schemes based on the even and odd ratios of the number of antennas per subarray *N* to the number of different phase shifts per symbol *K* (i.e., N/K), respectively. The simulation results show that the proposed approach greatly improves the estimation accuracy as compared to the state of the art when the ratio N/K is even.

## 1. Introduction

Due to the superior balance between performance and cost, a hybrid antenna array is regarded as an excellent candidate for millimeter wave (mmWave) communication systems [1,2]. Typically, the hybrid array is composed of multiple analog subarrays with phase controllable antenna elements. It includes two kinds of conventional configurations, i.e., localized and interleaved arrays in the light of the topology of subarrays. As the localized array is easier for schematic design and hardware implementation, it is more suitable for building a massive array. Angle-of-arrival (AoA) acquisition of the incoming mmWave signal is of considerable importance for signal reception, since the mmWave channels are dominated by the line-of-sight (LOS) propagation. A wide range of its applications including localization and tracking to mmWave communication systems, e.g., 5G mmWave cellular networks [2] and satellite-borne communications [1], have been increasingly studied in recent years.

AoA estimation using a localized array suffers from the phase ambiguity problem, which has been progressively studied in [3,4,5,6,7]. Each of these solutions leverages the cross-correlations between neighbouring subarrays for an AoA estimate. Phase ambiguity stems from an undetermined integer multiple of 2π between Nu and the argument of cross-correlations, where *N* is the number of antennas in a subarray, $u=\frac{2\pi }{\lambda }d\sin\theta$, θ the elevation AoA, λ the wavelength, and *d* the adjacent antenna spacing. With the identical phase shift deployment over all subarrays for constructive combination of cross-correlations, the work in [4] proposed a differential beam search algorithm to go through all possible beams and determine *u* with the largest output power. However, it incurs a long scanning period that linearly increases with the length of a subframe and *N*. To avoid a long scanning period, the authors in [5] studied a phase shift configuration with different values in different subarrays to eliminate the ambiguity by directly estimating *u*. Their ingenious idea is that (1) Nu is estimated by rectifying the signs of cross-correlations and then combining them coherently; (2) After calibrating subarray output signals with the estimated Nu, one takes their inverse discrete Fourier transform (IDFT) and calculates the correlations of the Fourier coefficients to uniquely recover *u*. The work in [3,6] further generalized the phase shift design in [5] to narrowband and wideband systems respectively, and revealed that the strongest cross-correlation takes the opposite sign from the remaining cross-correlations. Following this finding with an improved calibration accuracy of cross-correlations, the AoA can be speedily and reliably estimated even in low signal-to-noise ratio (SNR) regimes. In [7], an enhanced AoA estimation for a polarized mmWave signal was studied using a localized hybrid dual-polarized array, where polarization diversity combining was employed to improve the estimation of phase offset between adjacent subarrays. With the cross-correlation based algorithm, a multi-AoA estimation scheme with a combiner design was proposed in [8], where the paths for different users are identified by exploiting the low correlation property of the pseudo-random sequences.

With a digital array, MUSIC and ESPRIT [9] are the classical methods used in high-resolution AoA estimation. The work in [10,11] applied them to a localized array. Although accurate estimation can be achieved, the computational complexity incurred from singular value decomposition grows cubically with the total number of antennas [6], which makes the applications of these methods impractical in mmWave massive arrays. In [12], an auxiliary beam pair (ABP) design was proposed to provide high-resolution AoA estimation via amplitude comparison relating to each ABP. It, however, needs to scan all the directions of interest exhaustively, and the resolution is subject to the beamwidth and SNR. In [13], the optimal sum and difference beamformers based AoA estimator was constructed by exploiting the ratio of difference pattern to sum pattern with two overlapping subarrays, which can achieve the minimum estimation variance under Gaussian noise, regardless of any nulling performed. The work in [14] uses hierarchical search in the designed multi-resolution codebook to promptly identify one single multipath component (MPC) and thus the AoA. A compressed sensing based method was further investigated in [15] to find multiple MPCs, exploiting the sparse nature of mmWave channels. The beam needs to be recurrently narrowed down according to the codebook, which incurs additional overhead.

In this paper, we propose a novel phase shift design to enable unambiguous AoA estimation using a localized array. Instead of generating multiple single beams as proposed in [3,4,5,6,7], a difference beam based phase shift configuration is designed to steer each subarray in two directions. This can effectively improve the performance in terms of mean square error (MSE) of Nu estimation and detection probability of the expected subarray index by providing better coverage of the directions of interest. Based on the derivation in terms of the even and odd ratios of N/K where *K* is the different phase shifts per symbol, two IDFT-and-correlation based estimation schemes are proposed to directly estimate the AoA. Simulation results show the effectiveness of the proposed approach in estimation accuracy.

## 2. System Models

**Figure 1:**
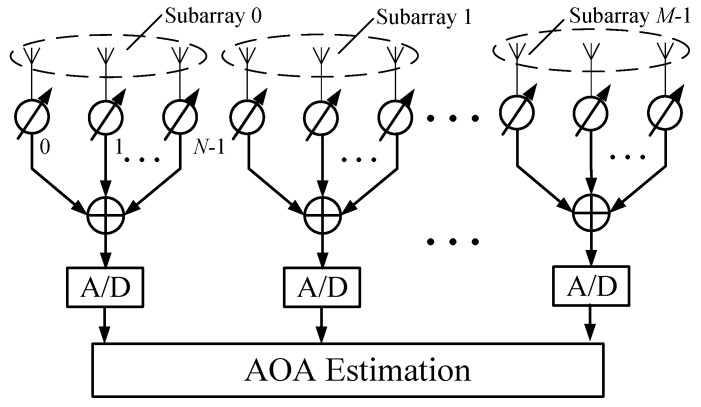
Illustration of a localized array with *M* subarrays, where the RF and down conversion components are omitted for simplicity.

As illustrated in Figure 1, we consider a uniform linear localized array composed of *M* subarrays, each with *N* evenly spaced phase-tunable antenna elements. Assume the arriving information-bearing signal $\tilde{s}\left(t\right)$ with wavelength λ and elevation angle θ. The received signals at the *m*th subarray (m=0,...,M−1) are combined after phase shifting, and then the analog beamformed signal is down-converted to baseband. Through analog-to-digital conversions, the output signal is given by [6]

(1) $$\begin{array}{c} s_{m}\left(t\right)=\tilde{s}\left(t\right)P_{m}\left(u,t\right)e^{jmNu}+\xi_{m}\left(t\right), \end{array}$$

where $\xi_{m}\left(t\right)$ is the zero-mean additive white Gaussian noise (AWGN) at the output of the *m*th subarray with power $\sigma_{n}^{2}$; $P_{m}\left(u,t\right)$ is the radiation pattern of the *m*th subarray at time *t* given by

(2) $$\begin{array}{c} P_{m}\left(u,t\right)=\sum_{n=0}^{N-1}{\overset{ˇ}{P}}_{m}^{n}\left(u\right)e^{j(nu+\alpha_{m}^{n}\left(t\right))}, \end{array}$$

where ${\overset{ˇ}{P}}_{m}^{n}\left(u\right)$ denotes the radiation pattern of the *n*th antenna element (n=0,...,N−1) at the *m*th subarray. As in [5,6], we assume ${\overset{ˇ}{P}}_{m}^{n}\left(u\right)=1$; $\alpha_{m}^{n}\left(t\right)$ represents the phase shift of the corresponding antenna element at time *t* and $u=\frac{2\pi }{\lambda }d\sin\theta$.

Let $\rho_{m}\left(t\right)$ denote the cross-correlation between the output signals of the *m*th and (m+1)th subarrays given by

(3) $$\begin{array}{cc} \rho_{m}\left(t\right)= & s_{m}^{*}\left(t\right)s_{m+1}\left(t\right)\\ = & |\tilde{s}{\left(t\right)|}^{2}P_{m}^{*}\left(u,t\right)P_{m+1}\left(u,t\right)e^{jNu}+{\tilde{s}}^{*}\left(t\right)P_{m}^{*}\left(u,t\right)e^{-jmNu}\xi_{m+1}\left(t\right)\\ \; & +\tilde{s}\left(t\right)P_{m+1}\left(u,t\right)e^{j(m+1)Nu}\xi_{m}^{*}\left(t\right)+\xi_{m}^{*}\left(t\right)\xi_{m+1}\left(t\right)\\ = & |\tilde{s}{\left(t\right)|}^{2}P_{m}^{*}\left(u,t\right)P_{m+1}\left(u,t\right)e^{jNu}+z_{m}\left(t\right), \end{array}$$

where ${\left(\cdot \right)}^{*}$ and |(·)| represent the conjugate and absolute value of (·), respectively; $z_{m}\left(t\right)$ is approximated as an AWGN.

In [4], identical phase shifts are used in all the subarrays, i.e., for any *m*, the values of $\alpha_{m}^{0}\left(t\right)$, ..., $\alpha_{m}^{N-1}\left(t\right)$ form the same arithmetic progression, such that Nu in (3) can be estimated by taking the argument of $\rho_{m}\left(t\right)$. However, since Nu can be outside the range [−π,π), the determination of *u* from the estimate of Nu ($\hat{Nu}$) will lead to phase ambiguity, i.e., there are 2⌊N/2⌋+1 possible estimates of *u*, given by $\hat{u}\left(p\right)=\frac{2\pi p+\hat{Nu}}{N}$, p=−⌊N/2⌋,−⌊N/2⌋+1,...,⌊N/2⌋, where ⌊·⌋ denotes the floor function. As a result, all possible directions need to be tested by applying a scanning beam within a long scanning frame, in order to find the one with the largest signal power, and thus incurring excessive delays.

## 3. Proposed AOA Estimation Approach

In this section, phase shifts providing difference beams are designed to facilitate the phase offset estimation between adjacent subarrays. Two AoA estimation schemes are proposed for direct AoA acquisition according to the value of N/K, where *K* is the number of different phase shifts for any symbol.

### 3.1. Phase Shift Design

Let the *n*th phase shift of the *m*th subarray at the *t*th (t=0,...,T−1) symbol be $\alpha_{m}^{n}\left(t\right)$ given by

(4) $$\begin{array}{c} \alpha_{m}^{n}\left(t\right)=\left\{\begin{array}{cc} & n\alpha_{m}\left(t\right),\;\;\;\;\;\;\;\;n=0,...,\frac{N}{2}-1\\ & \pi +n\alpha_{m}\left(t\right),\;\;n=\frac{N}{2},...,N-1 \end{array}\right. \end{array}$$

where $\alpha_{m}\left(t\right)=-2\pi \left(\;\mathrm{mod}\;\{m,K\}T+t\right)/L$, mod{·,·} represents the modulo operation, and thus mod{m,K} indicates that $\alpha_{m}^{n}\left(t\right)$ varies periodically every *K* subarrays in one symbol; *K* takes a value from (2,M] and N=QK, where *N* is assumed to be an even number and *Q* is an integer; *T* is the number of training symbols; L=TK is the total number of different phase shifts used in the system. The setting given by (4) is able to make the array scan potential 2L directions within [−π,π) across *T* symbols, ensuring that the AoA is acquired by at least one of the *L* beams with high gain. Note that it is necessary to have the mainlobes of two difference beams to cover the AoA so that sufficient energy can be obtained when computing the cross-correlation to estimate the AoA. According to the sampling theorem, at least *N* scanning beams are required to cover the AoA range of [−π,π) given the number of antennas per subarray *N*. Generally, the proper values of *N*, *K* and *T* are supposed to be set to satisfy N≤TK for good AoA coverage with beamforming gains.

**Figure 2:**
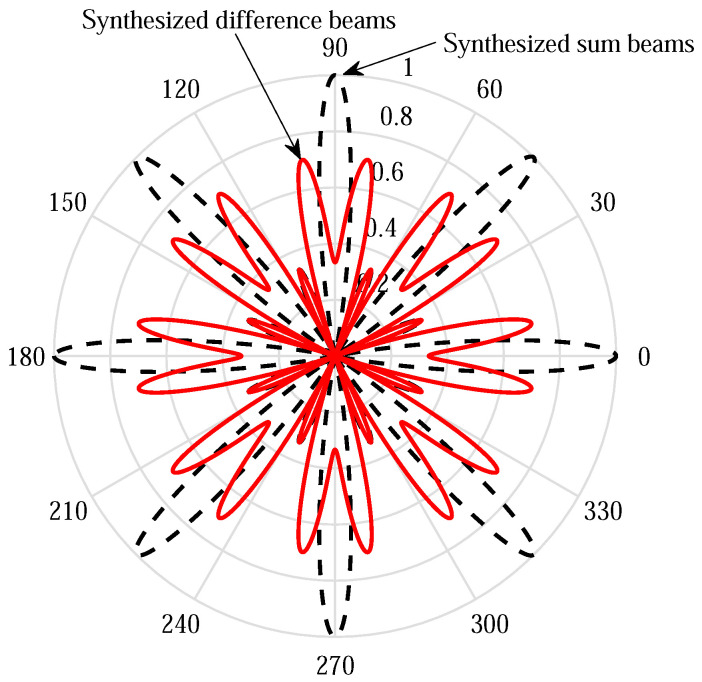
An example of normalized synthesized patterns of difference beams and sum beams.

Unlike the phase shift design in [6] that leverages multiple sum beams to steer multiple evenly distributed directions within [−π,π), the proposed one can steer double directions using each subarray by exploiting the difference beams [12]. Each difference beam steers a null towards the boresight of the corresponding sum beam. An example of normalized beam patterns within the first symbol period are shown in Figure 2, where the red solid and black dotted curves represent the synthesized difference beams and sum beams, respectively. In this example, we adopt K=M=8 and N=24, and therefore the phase shifter values of subarray *m* for difference beams are set to be −πmn/4 for n=0,...,11 and π(1−mn/4) for n=12,...,23, while for sum beams, −πmn/4 for n=0,...,23. When multiple training symbols are used, both null-steering direction and phase shifts are rotated by $\frac{2\pi }{L}$ between every two consecutive symbols. Although the maximal beamforming gain of a difference beam is 3 dB lower than that of a sum beam, multiple difference beams across multiple training symbols overlap in some directions of interest, which can make up for the beamforming gain loss.

### 3.2. Estimation of Nu

We apply Equation (4) to the estimation of Nu, which is then used to suppress $e^{jmNu}$ of $s_{m}\left(t\right)$ in (1) followed by the estimation of *u*. Substituting Equation (4) into Equation (2), we have

(5) $$\begin{array}{c} P_{m}\left(u,t\right)=\sum_{n=0}^{N/2-1}e^{jn(u+\alpha_{m}\left(t\right))}\left(1-e^{j\frac{N}{2}\left(u+\alpha_{m}\left(t\right)\right)}\right)=-2je^{j\left(N-1\right)\omega_{m}\left(u,t\right)}\frac{\sin^{2}\left(\frac{N\omega_{m}\left(u,t\right)}{2}\right)}{\sin(\omega_{m}\left(u,t\right))}, \end{array}$$

where $\omega_{m}\left(u,t\right)=\left(u+\alpha_{m}\left(t\right)\right)/2$. For convenience of illustration, we consider the first *K* subarrays even though the results apply to the remaining M−K subarrays. Therefore, $\omega_{m}\left(u,t\right)$ is simplified as $\omega_{m}\left(u,t\right)=\frac{u}{2}-\pi \left(\frac{m}{K}+\frac{t}{L}\right)$.

Substituting Equation (5) into Equation (3), $\rho_{m}\left(t\right)$ can be given by

(6) $$\begin{array}{c} \rho_{m}\left(t\right)={\left|\tilde{s}\left(t\right)\right|}^{2}G_{m}\left(u,t\right)e^{jNu}+z_{m}\left(t\right), \end{array}$$

where

$$\begin{array}{c} G_{m}\left(u,t\right)=4e^{\frac{-j(N-1)\pi }{K}}\frac{\sin^{2}\left(\frac{N\omega_{m}\left(u,t\right)}{2}\right)\sin^{2}\left(\frac{N\omega_{m+1}\left(u,t\right)}{2}\right)}{\sin\left(\omega_{m}\left(u,t\right)\right)\sin\left(\omega_{m+1}\left(u,t\right)\right)}. \end{array}$$

As specified in Lemma 1 [6], there exists a unique integer $m^{\prime }\in \left[0,K-1\right]$ satisfying $\sin\left(\omega_{m^{\prime }}\left(u,t\right)\right)\sin\left(\omega_{m^{\prime }+1}\left(u,t\right)\right)<0$. Given $m^{\prime }$, we have

(7) $$\begin{array}{cc} & 4\sin^{2}\left(\frac{N\omega_{m}\left(u,t\right)}{2}\right)\sin^{2}\left(\frac{N\omega_{m+1}\left(u,t\right)}{2}\right)\\ = & 1-\cos\left(N\omega_{m}\left(u,t\right)\right)-\cos\left(N\omega_{m+1}\left(u,t\right)\right)+\cos\left(N\omega_{m}\left(u,t\right)\right)\cos\left(N\omega_{m+1}\left(u,t\right)\right)\\ = & 1-2\cos\left(N\omega_{m^{\prime }}\left(u,t\right)+\frac{[2\left(m^{\prime }-m\right)-1]Q\pi }{2}\right)\cos\left(\frac{Q\pi }{2}\right)+{\left(-1\right)}^{[2\left(m^{\prime }-m\right)-1]Q}\cos^{2}\left(N\omega_{m^{\prime }}\left(u,t\right)\right), \end{array}$$

since $\omega_{m}\left(u,t\right)=\omega_{m^{\prime }}\left(u,t\right)+\frac{(m^{\prime }-m)\pi }{K}$. Considering two cases of *Q*, i.e., even and odd, we have

(8) $$\begin{array}{c} G_{m}\left(u,t\right)=\left\{\begin{array}{cc} & \frac{e^{j\frac{\pi }{K}}{\left[1-\cos\left(N\omega_{m^{\prime }}\left(u,t\right)\right)\right]}^{2}}{\sin\left(\omega_{m}\left(u,t\right)\right)\sin\left(\omega_{m+1}\left(u,t\right)\right)},\;\;\;\;\;Q\;\;\mathrm{is}\mathrm{even}\\ & \frac{-e^{j\frac{\pi }{K}}\sin^{2}\left(N\omega_{m^{\prime }}\left(u,t\right)\right)}{\sin\left(\omega_{m}\left(u,t\right)\right)\sin\left(\omega_{m+1}\left(u,t\right)\right)}.\;\;\;\;\;Q\;\;\mathrm{is}\mathrm{odd} \end{array}\right. \end{array}$$

Furthermore, as stated in Lemma 2 [6] that $|\sin\left(\omega_{m^{\prime }}\left(u,t\right)\right)\sin\left(\omega_{m^{\prime }+1}\left(u,t\right)\right)|$$<|\sin\left(\omega_{m}\left(u,t\right)\right)\sin\left(\omega_{m+1}\left(u,t\right)\right)|$, $\forall m\neq m^{\prime }$, only $G_{m^{\prime }}\left(u,t\right)$ with the largest amplitude has the opposite sign of the remaining since the numerator of $G_{m}\left(u,t\right)$ does not change with *m*. As a result, when the SNR is not very low, $m^{\prime }$ can be determined by $\rho_{m}\left(t\right)$ with the largest amplitude, i.e., $m^{\prime }=\underset{m=0:K-1}{\mathrm{argmax}}\left\{|\rho_{m}\left(t\right)|\right\}$. Given $m^{\prime }$, the signs of $\rho_{m}\left(t\right)$ (m=0,...,K−1) can be aligned following ${\tilde{\rho }}_{m}\left(t\right)={\left(-1\right)}^{Q+1\{m=m^{\prime }\}}\rho_{m}\left(t\right)$ for $\hat{Nu}$, where 1{·} is the indicator function. Note that, from the expression of $\alpha_{m}\left(t\right)$, we have $\rho_{m}\left(t\right)=\rho_{\;\mathrm{mod}\;\{m,K\}}\left(t\right)$ (m=0,...,M−2) while ignoring $z_{m}\left(t\right)$ in Equation (6), and hence the signs of $\rho_{m}\left(t\right)$ can be further calibrated following Step 7 in Algorithm 1. As shown in Step 9 of Algorithm 1, ${\tilde{\rho }}_{m}\left(t\right)$ across all subarrays and symbols can be coherently combined to improve the accuracy of $\hat{Nu}$.

### 3.3. Estimation of *u*

Next, we perform the estimation of *u* in terms of *Q* being even or odd as follows.

(I) When *Q* is even, letting n=k+qK, k=0,...,K−1, q=0,...,Q/2−1, Equation (5) can be written as

(9) $$\begin{array}{c} P_{m}\left(u,t\right)=\sum_{k=0}^{K-1}\sum_{q=0}^{Q/2-1}\left(1-e^{jN\omega_{m}\left(u,t\right)}\right)e^{j2\left(k+qK\right)\omega_{m}\left(u,t\right)}=\sum_{k=0}^{K-1}g_{k}\left(u,t\right)e^{-j\frac{2\pi mk}{K}}, \end{array}$$

where

$$\begin{array}{c} g_{k}\left(u,t\right)=-2j\frac{\sin^{2}\left(\frac{Nu}{4}-\frac{N\pi t}{2L}\right)}{\sin\left(\frac{Ku}{2}-\frac{K\pi t}{L}\right)}e^{j\left(N-K\right)\left(\frac{u}{2}-\frac{\pi t}{L}\right)}e^{jk(u-\frac{2\pi t}{L})} \end{array}$$

are the Fourier coefficients of $P_{m}\left(u,t\right)$.
**Algorithm 1** Estimation of Nu
**Input:** $s_{m}\left(t\right)$, m=0:M−1, t=0:T−1;
1:
**for** t=0:T−1  **do**
2:    Calculate $\rho_{m}\left(t\right)$ by (3), m=0:M−2;3:    **if** K=M **then**4:        $\rho_{K-1}\left(t\right)\leftarrow s_{K-1}^{*}\left(t\right)s_{0}\left(t\right)$;//The cross-correlation between the first and the last subarrays5:    **end if**6:    Determine $m^{\prime }\leftarrow \underset{m=0:K-1}{\mathrm{argmax}}\left\{|\rho_{m}\left(t\right)|\right\}$;//Find the subarray index with the largest amplitude7:    ${\tilde{\rho }}_{m}\left(t\right)\leftarrow {\left(-1\right)}^{Q+1\{\;\mathrm{mod}\;\left\{m-m^{\prime },K\right\}=0\}}\rho_{m}\left(t\right)$, m=0:M−2;//Calibrate their signs8:
**end for**
9:$\hat{Nu}\leftarrow \arg\left\{e^{-j\frac{\pi }{K}}\sum_{t=0}^{T-1}\sum_{m=0}^{M-2}{\tilde{\rho }}_{m}\left(t\right)\right\}$.//Coherent combination for improving estimation accuracy


Given $\hat{Nu}$, we calibrate $s_{m}\left(t\right)$ by multiplying $e^{-jm\hat{Nu}}$, i.e., $s_{m}\left(t\right)e^{-jm\hat{Nu}}$. Provided that $e^{jm(Nu-\hat{Nu})}\approx 1$, $s_{m}\left(t\right)$ can be almost perfectly calibrated. Performing the *K*-point IDFT of $s_{m}\left(t\right)e^{-jm\hat{Nu}}$ produces ${\tilde{S}}_{k}\left(t\right)\approx \tilde{s}\left(t\right)g_{k}\left(u,t\right)+\Xi_{k}\left(t\right)$, where $\Xi_{k}\left(t\right)$ are the *K*-point IDFT of $\xi_{m}\left(t\right)e^{-jm\hat{Nu}}$, m,k=0,...,K−1.

To obtain an estimate of *u*, $\hat{u}$, we compute the cross-correlation between any two adjacent IDFT outputs, ${\tilde{S}}_{k}^{*}\left(t\right){\tilde{S}}_{k+1}\left(t\right)$, denoted by $d_{k}\left(t\right)$, k=0,...,K−2, given by

(10) $$\begin{array}{c} d_{k}\left(t\right)=4{\left|\tilde{s}\left(t\right)\right|}^{2}\frac{\sin^{4}\left(\frac{Nu}{4}-\frac{N\pi t}{2L}\right)}{\sin^{2}\left(\frac{Ku}{2}-\frac{K\pi t}{L}\right)}e^{j(u-\frac{2\pi t}{L})}+{\tilde{\Xi }}_{k}\left(t\right), \end{array}$$

where ${\tilde{\Xi }}_{k}\left(t\right)$ is approximated as an AWGN. It is observed from Equation (10) that $\hat{u}$ can be unambiguously captured by $\hat{u}=\arg\left\{d_{k}\left(t\right)e^{\frac{j2\pi t}{L}}\right\}$. Similarly, $d_{k}\left(t\right)$ across all subarrays and symbols can be combined to improve the accuracy of $\hat{u}$.

(II) When *Q* is odd, *K* must be even since *N* is even. Letting n=k+qK/2, k=0,...,K/2−1, q=0,...,Q−1, (5) can be written as

(11) $$\begin{array}{c} P_{m}\left(u,t\right)=\sum_{k=0}^{K/2-1}\sum_{q=0}^{Q-1}\left(1-e^{jN\omega_{m}\left(u,t\right)}\right)e^{j\left(2k+qK\right)\omega_{m}\left(u,t\right)}=\sum_{k=0}^{K/2-1}\frac{{\left[1-{\left(-1\right)}^{m}e^{jN(\frac{u}{2}-\frac{\pi t}{L})}\right]}^{2}}{1-{\left(-1\right)}^{m}e^{jK(\frac{u}{2}-\frac{\pi t}{L})}}e^{jk(u-\frac{2\pi t}{L})}e^{-j\frac{2\pi mk}{K}}. \end{array}$$

Separating $P_{m}\left(u,t\right)$ to even and odd samples, we have

(12) $$\begin{array}{c} P_{m}\left(u,t\right)=\left\{\begin{array}{cc} & \sum_{k=0}^{K/2-1}\underset{g_{k}^{e}\left(u,t\right)}{\underset{︸}{\frac{{\left[1-e^{jN(\frac{u}{2}-\frac{\pi t}{L})}\right]}^{2}}{1-e^{jK(\frac{u}{2}-\frac{\pi t}{L})}}e^{jk(u-\frac{2\pi t}{L})}}}e^{-j\frac{2\pi lk}{K/2}},\;\;\;\;\;\;\;\;\;\;m=2l\\ & \sum_{k=0}^{K/2-1}\underset{g_{k}^{o}\left(u,t\right)}{\underset{︸}{\frac{{\left[1+e^{jN(\frac{u}{2}-\frac{\pi t}{L})}\right]}^{2}}{1+e^{jK(\frac{u}{2}-\frac{\pi t}{L})}}e^{jk(u-\frac{2\pi t}{L}-\frac{2\pi }{K})}}}e^{-j\frac{2\pi lk}{K/2}},\;m=2l+1 \end{array}\right. \end{array}$$

where l=0,...,K/2−1. Performing K/2-point IDFT of the even and odd samples of $s_{m}\left(t\right)e^{-jm\hat{Nu}}$, respectively, and then calculating the cross-correlation between adjacent IDFT outputs, denoted by $d_{k}^{e}\left(t\right)$ and $d_{k}^{o}\left(t\right)$, k=0,...,K/2−2, we have $\hat{u}$$=\arg\left\{d_{k}^{e}\left(t\right)e^{j\frac{2\pi t}{L}}+d_{k}^{o}\left(t\right)e^{j\left(\frac{2\pi t}{L}+\frac{2\pi }{K}\right)}\right\}$.

The estimation of *u* is summarized in Algorithm 2, where ${\tilde{S}}_{q}{\left(t\right)}_{\left(k_{1}:k_{2}\right)}$ denotes a vector consisting of the $k_{1}$th to $k_{2}$th elements of ${\tilde{S}}_{q}\left(t\right)$ and ${\left(\cdot \right)}^{T}$ stands for the transpose of (·). Note that in Step 12, the samples from the (M−⌊M/K⌋K)th to the (K−1)th are concatenated after the samples from the (⌊M/K⌋K)th to the (M−1)th to constructively estimate *u*, exploiting the periodicity of the phase shifts designed in (4). As the proposed approach is based on cross-correlation and IDFT computation, its computational complexity is similar to that in [6] given by $O(N\left(3+\log_{2}M\right))$, which is much lower than the subspace-based methods, e.g., MUSIC or ESPRIT in [10,11] given by $O(M^{3}N^{3})$.
**Algorithm 2** Estimation of *u*
**Input:** $\hat{Nu}$, $s_{m}\left(t\right)$, m=0:M−1, t=0:T−1;
1:
**for** t=0:T−1  **do**
2:    ${\tilde{s}}_{m}\left(t\right)\leftarrow s_{m}\left(t\right)e^{-jm\hat{Nu}}$, m=0:M−1;//Calibrate $s_{m}\left(t\right)$3:    **for** q=0:⌊M/K⌋−1 **do**//⌊M/K⌋ non-overlapping groups4:        **if** *Q* is even **then**5:           ${\tilde{s}}_{q}\left(t\right)\leftarrow \left\{{\tilde{s}}_{qK}\left(t\right),{\tilde{s}}_{qK+1}\left(t\right),...,{\tilde{s}}_{(q+1)K-1}\left(t\right)\right\}$,   ${\tilde{S}}_{q}\left(t\right)\leftarrow \mathrm{IDFT}\left\{{\tilde{s}}_{q}\left(t\right)\right\}$;//*K*-point IDFT6:        **else**7:           ${\tilde{s}}_{q}^{e}\left(t\right)\leftarrow \mathrm{the}\;\mathrm{even}\;\mathrm{samples}\;\mathrm{of}\;\;{\tilde{s}}_{q}\left(t\right)$,   ${\tilde{S}}_{q}^{e}\left(t\right)\leftarrow \mathrm{IDFT}\left\{{\tilde{s}}_{q}^{e}\left(t\right)\right\}$;//K/2-point IDFT8:           ${\tilde{s}}_{q}^{o}\left(t\right)\leftarrow \mathrm{the}\;\mathrm{odd}\;\mathrm{samples}\;\mathrm{of}\;\;{\tilde{s}}_{q}\left(t\right)$,   ${\tilde{S}}_{q}^{o}\left(t\right)\leftarrow \mathrm{IDFT}\left\{{\tilde{s}}_{q}^{o}\left(t\right)\right\}$;//K/2-point IDFT9:        **end if**10:    **end for**11:    **if** *Q* is even **then**12:        ${\tilde{s}}_{\left\lfloor M/K\right\rfloor }\left(t\right)\leftarrow \left\{{\tilde{s}}_{\lfloor M/K\rfloor K}\left(t\right),...,{\tilde{s}}_{M-1}\left(t\right),{\tilde{s}}_{M-\lfloor M/K\rfloor K}\left(t\right),...,{\tilde{s}}_{K-1}\left(t\right)\right\}$;13:        ${\tilde{S}}_{\left\lfloor M/K\right\rfloor }\left(t\right)\leftarrow \mathrm{IDFT}\left\{{\tilde{s}}_{\left\lfloor M/K\right\rfloor }\left(t\right)\right\}$;14:    **else**15:        ${\tilde{s}}_{\left\lfloor M/K\right\rfloor }^{e}\left(t\right)\leftarrow \mathrm{the}\;\mathrm{even}\;\mathrm{samples}\;\mathrm{of}\;\;{\tilde{s}}_{\left\lfloor M/K\right\rfloor }\left(t\right)$,   ${\tilde{S}}_{\left\lfloor M/K\right\rfloor }^{e}\left(t\right)\leftarrow \mathrm{IDFT}\left\{{\tilde{s}}_{\left\lfloor M/K\right\rfloor }^{e}\left(t\right)\right\}$;16:        ${\tilde{s}}_{\left\lfloor M/K\right\rfloor }^{o}\left(t\right)\leftarrow \mathrm{the}\;\mathrm{odd}\;\mathrm{samples}\;\mathrm{of}\;\;{\tilde{s}}_{\left\lfloor M/K\right\rfloor }\left(t\right)$,   ${\tilde{S}}_{\left\lfloor M/K\right\rfloor }^{o}\left(t\right)\leftarrow \mathrm{IDFT}\left\{{\tilde{s}}_{\left\lfloor M/K\right\rfloor }^{o}\left(t\right)\right\}$;17:    **end if**18:
**end for**
19:**if***Q* is even **then**20:    $\hat{u}\leftarrow \arg\left\{\sum_{t=0}^{T-1}e^{j\frac{2\pi t}{L}}\sum_{q=0}^{\left\lfloor M/K\right\rfloor }{{\tilde{S}}_{q}^{*}\left(t\right)}_{\left(1:K-1\right)}{{\tilde{S}}_{q}^{T}\left(t\right)}_{\left(2:K\right)}\right\}$;//Coherent combination21:
**else**
22:    $\hat{u}\leftarrow \arg\left\{\sum_{t=0}^{T-1}e^{j\frac{2\pi t}{L}}\sum_{q=0}^{\left\lfloor M/K\right\rfloor }\left({{\tilde{S}}_{q}^{e*}\left(t\right)}_{\left(1:\frac{K}{2}-1\right)}{{\tilde{S}}_{q}^{eT}\left(t\right)}_{\left(2:\frac{K}{2}\right)}+e^{j\frac{2\pi }{K}}{{\tilde{S}}_{q}^{o*}\left(t\right)}_{\left(1:\frac{K}{2}-1\right)}{{\tilde{S}}_{q}^{oT}\left(t\right)}_{\left(2:\frac{K}{2}\right)}\right)\right\}$.23:
**end if**



### 3.4. Discussion on Extension of the Proposed Approach

The proposed approach can be potentially extended to wideband mmWave systems, where each subcarrier or a cluster of subcarriers are assumed to be narrowband, and the proposed approach is performed separately at different subcarriers or clusters. The cross-correlations between subcarriers or clusters can also be exploited to improve the estimation accuracy [3].

The proposed approach can be extended from a linear array to a planar array, where the proposed phase shift design and cross-correlation operation can be applied similarly along the orthogonal dimension. Since the radiation pattern of a planar array can be represented by the product of independent radiation patterns along two orthogonal dimensions, the AoA estimation between them can be decoupled from each other.

The proposed approach can potentially be extended to the case in the presence of nonline-of-sight (NLOS) or interferences from other transmitters. Since the NLOSs are typically much weaker than the LOS, serial interference cancellation could be performed for sequential AoA estimation with the proposed approach. When the AoA of the LOS is estimated, we can steer all beams of subarrays towards this direction, and then evaluate its channel amplitude and phase. By regenerating the LOS signal component and removing it from the received signals in all subarrays, the second strongest path can be estimated. In the same way, the remaining paths can be estimated and subtracted one by one. When there exist multiple interferences with similar power from different directions, the proposed approach could be conducted in terms of parallel interference cancellation, where multiple AoAs are simultaneously estimated and cancelled.

## 4. Simulation Results

In this section, we present the simulation results to evaluate the proposed approach, compared with the state of the art [6]. Denote the average received SNR per antenna as $\gamma_{a}$. The training symbols, $\tilde{s}\left(t\right)$, are generated following complex Gaussian distributions with zero mean. Assuming uniformly distributed AoA within [−π,π], simulation results are obtained by averaging over 50,000 trials. Here, we define $P_{d}$ as the probability of correctly finding the index $m^{\prime }$ at Step 6 of Algorithm 1.

**Figure 3:**
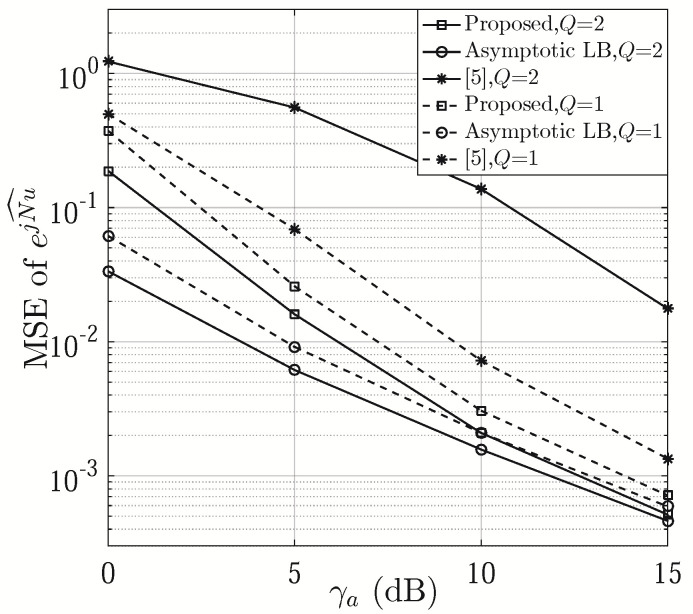
MSE of $e^{j\hat{Nu}}$ versus $\gamma_{a}$ (M=K=8 and T=16).

Figure 3 compares the MSEs of $e^{j\hat{Nu}}$ versus $\gamma_{a}$ with Q=1 and 2. As shown in the figure, the proposed phase shift design outperforms that of [6] in terms of MSE of $e^{j\hat{Nu}}$, since a higher SNR for $\hat{Nu}$ can be achieved at Step 9 of Algorithm 1. The MSE curve of $e^{j\hat{Nu}}$ becomes increasingly tight to its asymptotic lower bound with the increase of $\gamma_{a}$, where the asymptotic lower bound is the lower bound of proposed approach produced under the assumption of $P_{d}=1$. There is more gain with Q=2 than Q=1 in comparison with [6], which indicates that the proposed scheme is more applicable to narrow beams, i.e., large *N*. This is because multiple narrower single beams cannot provide desirable AoA coverage, resulting in estimation performance loss, which, however, can be compensated by our phase shift design.

**Figure 4:**
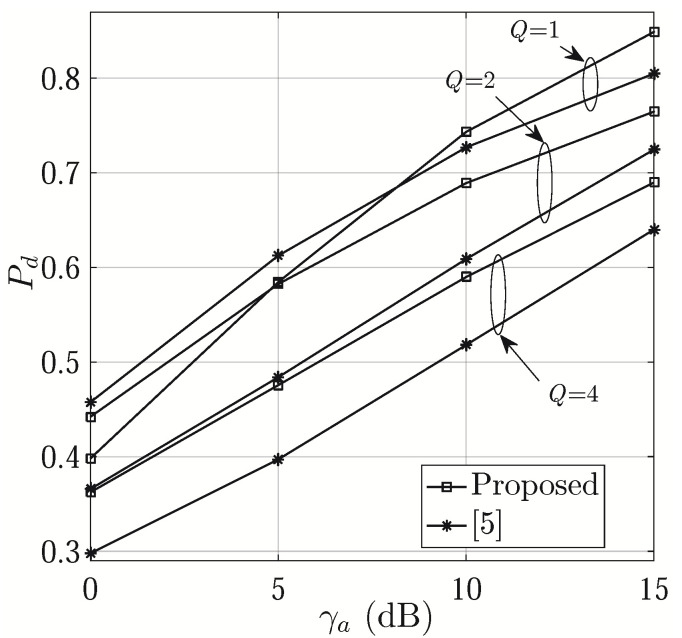
$P_{d}$ versus $\gamma_{a}$ (M=K=8 and T=16).

Figure 4 shows $P_{d}$ versus $\gamma_{a}$ with different values of *Q*. We can see that the proposed scheme generally has better performance. At high SNRs, it achieves higher $P_{d}$ with Q=1, while it is inferior to that of [6] when SNR is low. Note that, compared with that in [6], our proposed phase shift design improves the capability of identifying the correct $m^{\prime }$, thus effectively suppressing the noise and indirectly improving the SNR of estimation. When $\gamma_{a}$ is greater than 5 dB, the proposed one leads to a higher $P_{d}$ with a smaller *Q*. This is because, when the number of beams is fixed within one symbol period, a smaller *N*, and hence a wider beam, leads to a better coverage of the directions of interest. Therefore, it is easier to find the correct $m^{\prime }$.

**Figure 5:**
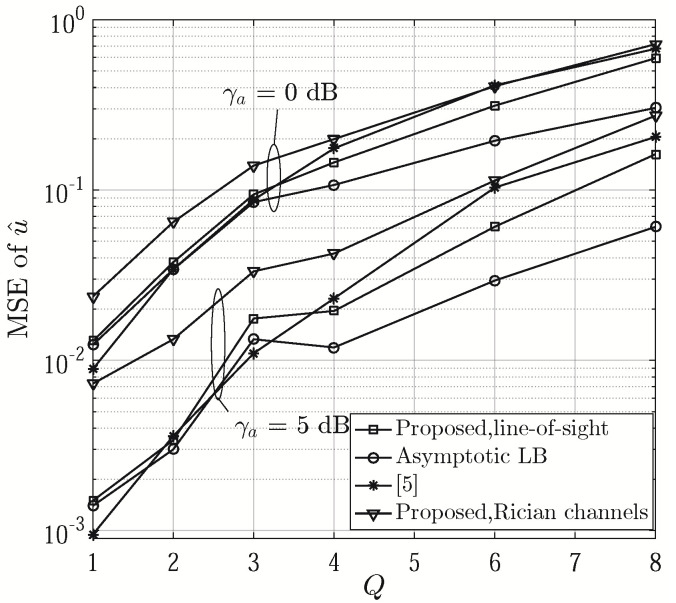
MSE of $\hat{u}$ versus *Q*, (N=24 and T=6).

**Figure 6:**
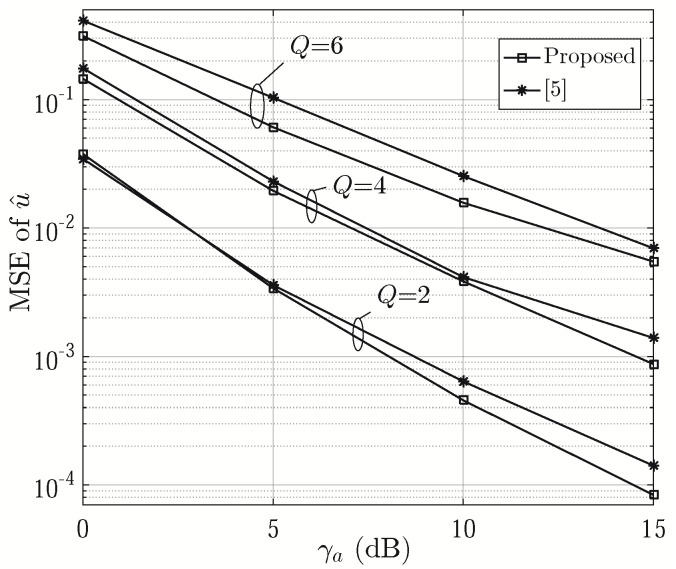
MSE of $\hat{u}$ versus $\gamma_{a}$, (N=24 and T=6).

The MSEs of $\hat{u}$ are shown as a function of *Q* in Figure 5. It can be seen that its MSEs generally increase with *Q* attributed to the decreasing number of subarrays. The proposed approach generally achieves better performance than [6] when *Q* is an even number. When *Q* is odd, the proposed method results in larger estimation errors since the signals are only averaged over K−2 product terms (see Step 22 of Algorithm 2), less than K−1 in [6]. The corresponding asymptotic lower bound of MSEs of $\hat{u}$ are displayed for comparison. To evaluate the credibility of estimation errors, we calculate 95% confidence intervals (CIs) for $\hat{u}$. When $\gamma_{a}=5$ dB and Q=2, 4 and 6, the CIs are given by [–0.0207, 0.0111], [–0.0204, 0.0114] and [–0.0244, 0.0075] for the proposed one, [–0.0219, 0.0099], [–0.0258, 0.0060] and [–0.0224, 0.0096] for [6], [–0.0205, 0.0113], [–0.0232, 0.0086] and [–0.0213, 0.0104] for the asymptotic lower bound, respectively. The MSEs of $\hat{u}$ in Rician fading channels [12] are also provided to show the impact of multipath channels on the proposed approach, where the Rician factor is assumed to be 10 dB. Figure 6 presents the MSE of $\hat{u}$ versus $\gamma_{a}$ with even values of *Q*. From the figure, we can see that the proposed approach outperforms [6] by 1.4 dB at the MSE of 0.1, 0.4 dB at the MSE of 0.01 and 0.7 dB at the MSE of 0.001, respectively, when *Q* = 6, 4 and 2.

## 5. Conclusions

In this letter, we proposed a novel phase shift design to facilitate the estimation of a single AoA in a localized hybrid array. Based on the ratio of the number of antennas per subarray to the number of different phase shifts per symbol being even or odd, we presented two different AoA estimation schemes. Employing difference beam steering in each subarray, the proposed approach can effectively improve the phase offset estimate accuracy between adjacent subarrays, and thus the AoA estimate. Simulation results of MSEs showed that the proposed one achieved better AoA estimation performance over the state of the art when *Q* is designed to be an even number.

## Data Availability

Not applicable.
